# Safety and efficacy of OAGB/MGB during the learning curve: setting a benchmark in a bariatric center of excellence

**DOI:** 10.1007/s13304-022-01380-9

**Published:** 2022-09-28

**Authors:** Mario Musella, Giovanna Berardi, Nunzio Velotti, Vincenzo Schiavone, Cristina Manetti, Antonio Vitiello

**Affiliations:** Advanced Biomedical Sciences Department, Naples “Federico II” University, AOU “Federico II”, Via S. Pansini 5, 80131 Naples, Italy

**Keywords:** One anastomosis gastric bypass, Mini-gastric bypass, Surgical training, Learning curve, Global benchmarks, Complications

## Abstract

Very little has been published on the learning curve (LC) of the One Anastomosis /Mini Gastric Bypass (OAGB/MGB). Aim of this study was to compare perioperative outcomes of OABG/MGBs performed during the LC of an experienced laparoscopic surgeon to global benchmark cut-offs. First 200 patients undergoing OAGB/MGB at our university hospital from 2010 to 2016 were retrospectively included in this study. LC of the surgeon was divided in two groups of 100 consecutive patients each and perioperative outcomes were compared to abovementioned global benchmarks for LSG and RYGB. A cumulative sum (CUSUM) analysis was performed for operative time and hospital stay. Uneventful postoperative recovery was recorded in 95% of patients. All benchmark values for RYGB were met in group 2. Comparison with cut-offs for LSG showed longer hospital stay and operative time in both groups but postoperative rate of complications resulted lower even for Group 1. CUSUM graph of the operative time runs randomly above the predetermined limit till the 40th cases but reaches the plateau after the 115th operation. CUSUM curve of the hospital stay reaches the plateau after the 57th case. OAGB/MGB confirms to be a feasible procedure, which can be safely and effectively performed during the learning curve. However, at least 100 hundred cases are required to reduce operative time and hospital stay.

## Introduction

In 1967, Mason first introduced a gastric bypass with one anastomosis for the treatment of morbid obesity [1]. Later, in 1997, Rutledge introduced a totally different version of a single anastomosis gastric bypass, which he named the Mini-Gastric Bypass (MGB). This intervention was significantly different from the one proposed by Mason since it consisted of one anastomosis between a long-sleeved gastric pouch and a jejunal loop [2].

An initial strong opposition came from authoritative surgeons against MGB, which was wrongly considered similar to the Mason’s bypass and the Billroth II resection [3].

The MGB or One Anastomosis Gastric Bypass (OAGB) [4] has shown so far to be extremely effective in inducing weight loss and reducing obesity-related comorbidities [5, 6]. It has been recently recognized by the American Society for Metabolic and Bariatric Surgery (ASMBS) [7] and IFSO reports show that the trend of MGB/OAGB is rapidly growing [8].

Compared to the traditional Roux-en-Y Gastric Bypass (RYGB), the OAGB/MGB induces better results with a simpler surgical technique and shorter operative time [9, 10]

Despite this feasibility, OAGB/MGB remains a laparoscopic gastric bypass procedure that requires an appropriate learning curve (LC) to reduce perioperative complications [11]. Several articles have investigated the minimum number of cases required to reach a significant reduction in operative time and morbidity after RYGB [12, 13] and after LSG (Laparoscopic Sleeve Gastrectomy) [14, 15], but very little has been published on the LC of the OAGB/MGB. The precise number of OAGB/MGBs required to achieve proficiency is still matter of debate.

Recently, global benchmarks for LSG and RYGB were set as the 75th percentile of morbidity in 19 high-volume academic centres in 3 continents: below this value perioperative outcomes are considered acceptable [16].

Aim of this study was to compare perioperative outcomes of the first 200 cases of OABG/MGBs performed at our institution to recently introduced global benchmarks values.

## Methods

First 200 patients undergoing OAGB/MGB at our university from January 2010 to December 2016 were included in this study.

Indications for surgery followed the recommendations of the International Federation of Surgery for Obesity and Metabolic Disorders (IFSO) [17].

Since the main inclusion criteria was the chronological order, also patients with a body mass index (BMI) > 50 kg/m^2^ or with a previous history of bariatric or abdominal surgery were included.

All the procedures were performed by the same surgeon who had previously completed the learning curve of index bariatric procedures.

LC of the surgeon was divided in two groups (Group 1 and 2) of 100 consecutive patients each and perioperative outcomes were compared to abovementioned global benchmarks for LSG and RYGB. Data on preoperative demographics (gender, age, comorbidities, Body Mass Index—BMI and history of previous bariatric surgery, number of subjects with BMI > 50), perioperative data (operative time, conversion to open, use of staple-line reinforcement, reoperation rate, length of hospital stay, readmissions, intra- and post-operative complications, mortality) were registered. Weight loss was calculated at 1.6 and 12 months as change of BMI and percentage of total weight loss (%TWL) using the following formula:

$$[{\text{initial}}\,{\text{weight}} - {\text{final}}\,{\text{weight}}/{\text{initial}}\,{\text{weight}}] \times 100$$

Postoperative complications were classified in accordance with the Clavien–Dindo classification [18].

The present research was approved by the institutional review board of our Department and informed consent to surgery was obtained from all patients.

### Surgical technique

Standard technique for OAGB/MGB has been previously reported [19, 20]: a six-port (5 × 10 mm, 1 × 5 mm) approach was used. The gastric pouch was fashioned along a 36-Fr starting just below the crow’s foot. No reinforcement was routinely applied on the staple line. Initially the biliopancreatic limb (BPL) had a fixed length of 200 cm, but after the first cases BPL was tailored on the patient’s BMI (Body Mass Index) [21]. The gastrojejunostomy was performed using a 45-mm linear stapler and enterotomies were closed by an anterior, double-layer, self- locking, running absorbable suture (V-lock 3/0, Medtronic™, Minneapolis, U.S.A.). Upper endoscopy is not used in our institution to check the anastomosis, but a methylene blue test is performed.

The nasogastric tube was removed the evening of the surgery and an abdominal drain was routinely placed behind the anastomosis. A liquid diet was started on postoperative day 3 and discharge was scheduled in case of no clinical signs of leak or stenosis.

### Statistical analysis

Data are expressed as mean ± DS. Two-tailored *t* test was used to compare continuous variables as appropriate, while categorical data were compared using the chi-square or Fisher’s exact test. Significant *p* value was set below 0.05.

A cumulative sum (CUSUM) analysis was performed for operative time and hospital stay [22, 23]. Results were presented in CUSUM charts which are a graphical presentation of the outcomes of a series of consecutive procedures. During the LC, the CUSUM curve runs above a decision interval when an operation is performed at an unacceptable level. The intervals were set according to global benchmark values for RYGB (duration of the operation = 120 min; hospital stay = 4).

## Results

### Baseline characteristics

Data on 200 consecutive patients were collected and included in the present study; female/male ratio was 41/159 and mean preoperative age and BMI were 42 ± 10 years and 44.8 ± 6.4 kg/m^2^ respectively. Thirty subjects had previously undergone a bariatric procedure (7 gastric band, 23 LSG) and 20 had a previous history of abdominal surgery (4 cesarean sections, 10 laparoscopic cholecystectomy, 5 umbilical hernia repairs, 1 appendectomy). Demographics and rate of patients with previous surgery were comparable preoperatively in the two groups (Table 1). Group 1 represents the first 100 cases and Group 2 the second 100 patients.

**Table 1:** Comparison of demographics in the two groups of patients

Parameter	Group 1 (*n* = 100)	Group 2 (*n* = 100)	*p* value
Age (years) BMI (Kg/m^2^)	45.1 ± 6.5	44.5 ± 6.3	0.46
Sex (F/M)	16/84	25/75	0.11
Age (years)	43.9 ± 9.6	41.9 ± 10.3	0.53
Previous bariatric surgery (*n*, %)	11 (11%)	19 (19%)	0.11
Previous abdominal surgery (*n*, %)	7 (7%)	13 (13%)	0.15
Patients with BMI > 50	15 (15%)	18 (18%)	0.56

Group 1 = OAGB/MGB cases from 1 to 100; Group 2 = OAGB/MGB cases from 100 to 200

### Weight loss

**Fig. 1:**
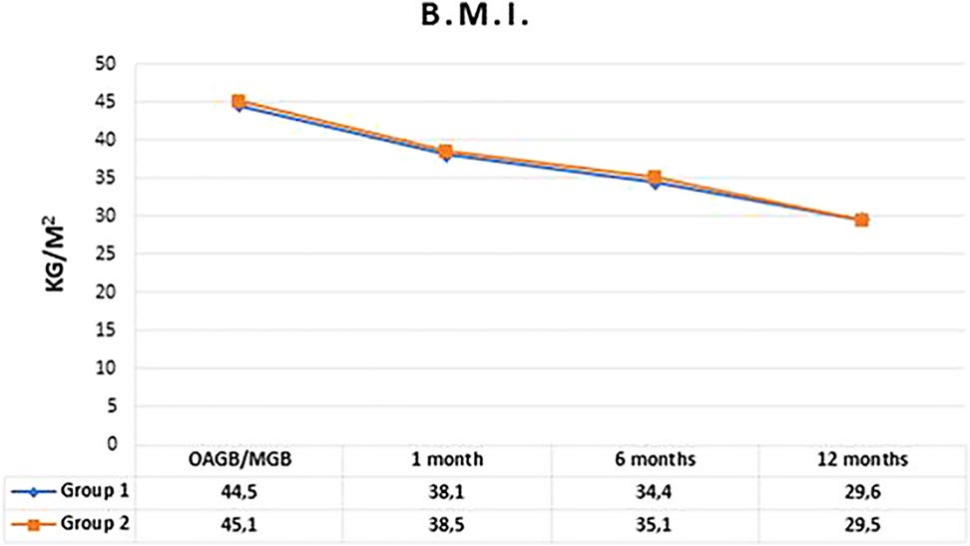
Trend of BMI in the two groups in the first 12 months. *p* value was 0.61, 0.34 and 0.86, respectively

**Fig. 2:**
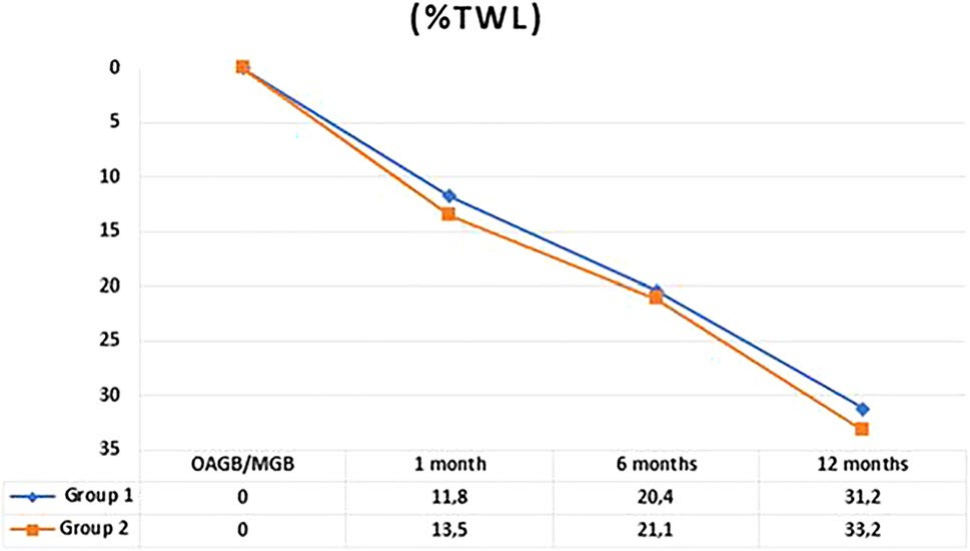
Trend of %TWL in the two groups in the first 12 months. *p* value was 0.16, 0.56 and 0.21, respectively

Mean BMI at 1.6 and 12 months after OAGB/MGB was 38.3 ± 5.2, 34.7 ± 4.8 and 29.6 ± 4.9 respectively. Percentage of total weight loss (%TWL) was 12.7 ± 8.5 after 1 month, 20.8 ± 9.0 at 6 months and 32.2 ± 10.9 after 12 months. No statistical difference in BMI and %TWL was found between the two groups at any time of follow-up (Figs. 1, 2). Follow-up rate at 1,6 and 12 months were 100%,98% and 90% in Group 1 and 100%, 100% and 99% in Group 2.

### Complications and comparison with global benchmarks

Mean operative time was 113.1 ± 30.9 min and hospital stay was 3.7 ± 0.8 days as per our standard protocol of discharge. Three patients had postoperative bleeding requiring transfusion (2 from trocars’ site and 1 from the anastomosis) and one subject was readmitted due to mild melena successfully treated without transfusion. One case of reoperation occurred in Group 1 due to small bowel injury. No case was converted to open surgery and no leak was recorded. Uneventful postoperative course was recorded in 95% of patients. Comparison between two groups shows a significant reduction in hospital stay and operative time after the first 100 OAGB/MGBs (*p* < 0.05, Tables 2 and 3). As reported in Tables 2 and 3, all benchmark values for RYGB were met in group 2. Comparison with cut-offs for LSG showed longer hospital stay and operative time in both groups but postoperative rate of complications resulted lower even for Group 1.

**Table 2:** Comparison of perioperative complications in the two groups with global benchmarks

Perioperative complications	Benchmark cut-offs (75th Percentile) RYGB	Benchmark cut-offs (75th Percentile) LSG	Group 1	Group 2	*p* value
Operation duration (min)	120	90	131.6 ± 30.1	94.4 ± 17.7	< 0.0001
Conversion to open	0%	0%	0	0	1
Intraoperative blood transfusion	0%	0%	0	0	1
Postoperative blood transfusion	2%	1.3%	(3) 3%	(0) 0%	0.24
Postoperative ICU admission	0.14%	0%	(1) 1%	0%	1
ICU stay in patients admitted to ICU (days)	1	4	2	0	1
Hospital stay	4	3	3.9 ± 0.9	3.5 ± 0.5	0.0003

**Table 3:** Comparison of postoperative complications (< 90 days) in the two groups with global benchmarks

Perioperative complications until 90 days	Benchmark cut-offs (75th Percentile) RYGB	Benchmark cut-offs (75th Percentile) LSG	Group 1	Group 2	*P* value
Uneventful postoperative course	> 90%	> 88%	(97) 97%	(98) 98%	1
Readmission	< 5.5%	< 5.5%	(0)0%	(1) 1%	1
Reoperation	< 4%	< 3%	(1) 1%	0%	1
Any complication	< 10%	< 12%	(3) 3%	2 (2%)	1
Complication grade > IIIa	< 5.5%	< 5.5%	1%	0%	1
Mortality	0%	0%	0	0	1
anastomotic leak	< 1.3%	< 0.15%	0	0	1
Stenosis	< 1.2%	0%	0	0	1
Postoperative bleeding	< 2.2%	(3) 6%	(2) 2%	(1) 1%	1
Small bowel obstruction/internal hernia	< 0%	0%	0%	0%	1
Marginal ulcer	< 0%	/	0%	0%	1

### CUSUM analysis of the learning curve

**Fig. 3:**
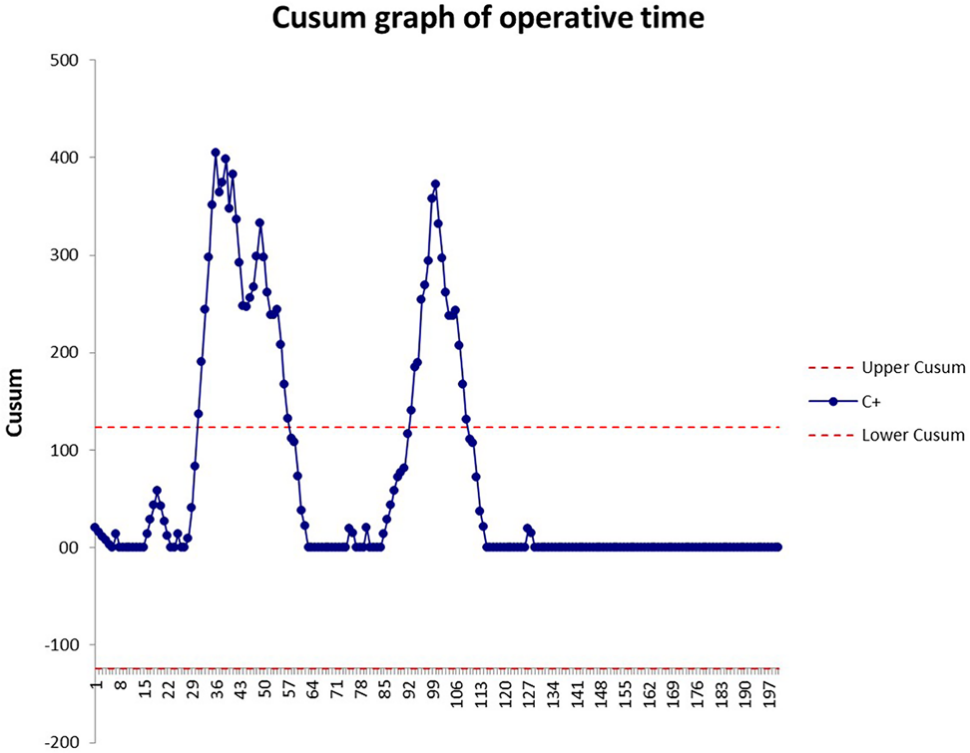
CUSUM curve of operative time

CUSUM graph of the operative time (Fig. 3) runs randomly above the predetermined limit till the 40^th^ cases but reaches the plateau after the 115th operation.

**Fig. 4:**
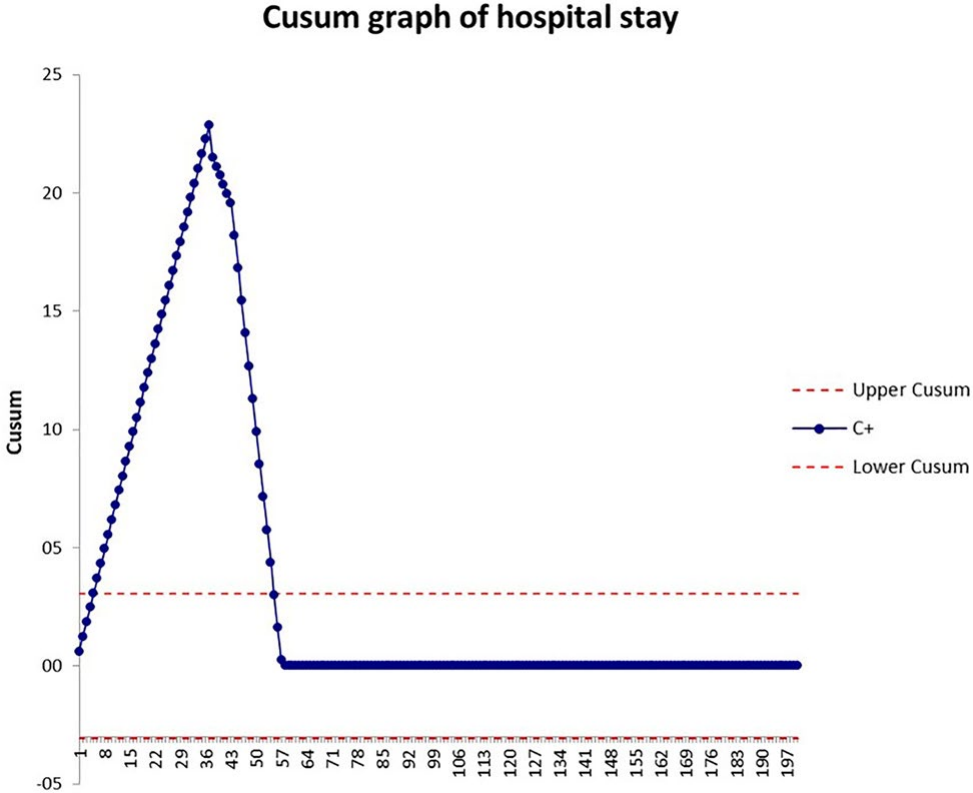
CUSUM curve of hospital stay

CUSUM curve of the hospital stay already reaches the plateau after the 57th case (Fig. 4).

## Discussion

Our experience shows that, in the first hundred cases, outcomes of OAGB/MGB mostly fall within internationally accepted rates of perioperative complications. The only significant improvement after the first 100 cases was the reduction of operative time and hospital stay. Indeed, the plateau of operative time was reached only after the first 100 cases, and the mean duration of the intervention in Group 2 was 94.4 ± 17.7 min, which was comparable to previously reported data in large series [24, 25].

Even if Early Recovery After Surgery (ERAS) pathway significantly reduces length of stay and cost after OAGB/MGB[26], this protocol of discharge does not apply to our hospital due to the absence of a Trauma and Emergency admission service. Nevertheless, the CUSUM graph showed an early reduction of hospital stay.

Safety of OAGB/MGB during the learning curve appears even more impressive if we consider that these global cut-offs were defined including only patients without previous abdominal surgery and excluding individuals with BMI > 50 kg/m^2^, while we did not adopt these safety criteria.

Correct surgical technique is also important to obtain satisfactory weight loss; there is a consensus [27] that the biliopancreatic limb length needs to be tailored to optimize bariatric results and avoid excessive malabsorption. The ideal BPL length is still matter of discussion and many authors suggest a routinely total bowel measurement to leave a common limb 300–400 cm long [28, 29]. Measurement of the entire intestine in a patient with severe obesity is a challenging task that can lead to bowel injuries, but so far reports of this complication after OAGB/MGB are rare and only one case (0.5%) occurred in our first group of patients. Moreover, weight loss was comparable in the two groups at any time of follow-up, demonstrating that lengths of BPL and common limb was correctly chosen also during the learning curve.

Despite no case of marginal ulcer occurred during the first 90 days in the two groups, recently a laparoscopic conversion to RYGB was carried out for a late (> 90 days) perforation [30].

### Strength and limitation

To the best of our knowledge, the present study is the only study investigating LG of OAGB/MGB with a large cohort of patients and comparison to global benchmark values.

Retrospective nature and previous experience with bariatric surgery may have biased results; probably complication rate would be higher for newly trained surgeons.

We used international cut-offs for RYGB, specific values for OAGB are not available and should be defined.

## Conclusion

OAGB/MGB confirms to be a feasible procedure, which can be safely and effectively performed during the learning curve. However, at least 100 hundred cases are required to reduce operative time and hospital stay.
